# Diagnostic Delay and Clinical Follow-Up Across Oral Lichen Planus Phenotypes: A Retrospective Cohort Study

**DOI:** 10.3390/jcm15166309

**Published:** 2026-08-14

**Authors:** Keren Martí De Gea, Massimo Petruzzi, Pia López-Jornet

**Affiliations:** 1 Dentistry Clinic, Department of Dermatology, Stomatology, Radiology and Physical Medicine, Faculty of Medicine, Hospital Morales Meseguer, University of Murcia, Marques Velez S/N, 30008 Murcia, Spain; k.martigea@um.es; 2 Department of Odontostomatology and Surgery, University of Bari, 70124 Bari, Italy; massimo.petruzzi@uniba.it

**Keywords:** oral lichen planus, diagnostic delay, diagnostic interval, oral potentially malignant disorders, clinical follow-up, oral medicine

## Abstract

**Background:** Oral lichen planus is a chronic oral potentially malignant disorder requiring timely diagnosis and long-term follow-up, yet its diagnostic pathway remains poorly characterised. This study assessed diagnostic and follow-up intervals in patients with oral lichen planus and explored associated clinical and healthcare-related factors. **Methods:** This retrospective cohort study included 199 patients with clinically and histopathologically confirmed oral lichen planus managed at the Oral Medicine Unit of the University of Murcia. Diagnostic time points were defined using an adapted Aarhus framework. Patient, primary care, specialist diagnostic and total intervals were analysed using multivariable quasi-Poisson regression. Loss to follow-up was assessed using Cox regression. **Results:** Older age was associated with longer patient, primary care and total intervals. Women had a shorter patient interval than men (IRR = 0.44; 95% CI: 0.21–0.91; *p* = 0.03), but a longer primary care interval (IRR = 3.09; 95% CI: 1.12–10.2; *p* = 0.04). Compared with erosive oral lichen planus, mixed and reticular forms showed shorter primary care intervals and shorter specialist diagnostic intervals, with IRRs ranging from 0.21 to 0.36 (all *p* < 0.01). Daily alcohol consumption was associated with the longest total interval. Older age was associated with a higher risk of loss to follow-up (HR = 1.02; 95% CI: 1.00–1.04; *p* = 0.01), whereas non-pharmacological treatment was associated with a lower hazard of loss to follow-up (HR = 0.31; 95% CI: 0.16–0.60; *p* < 0.001). **Conclusions:** Diagnostic delay in oral lichen planus is multifactorial and appears to depend on patient characteristics, clinical presentation and healthcare pathway factors. Improved recognition of erosive disease, clearer referral criteria and structured follow-up strategies may enhance care continuity.

## 1. Introduction

Oral potentially malignant disorders (OPMDs) comprise a heterogeneous group of oral mucosal alterations associated with an increased risk of transformation into oral squamous cell carcinoma. Among them is oral lichen planus (OLP), a chronic inflammatory disease of probable autoimmune origin characterised by a fluctuating clinical course [1].

OLP may present with different clinical patterns, including reticular, atrophic, and erosive forms, which can sometimes make it difficult to distinguish from other oral mucosal diseases. Reticular forms are generally the most frequent and are often asymptomatic, whereas atrophic, erosive, and ulcerative forms are more commonly associated with pain, functional impairment, and a negative impact on patients’ quality of life. Furthermore, owing to their potential for malignant transformation, these patients require periodic clinical surveillance and long-term follow-up [2,3,4].

At present, there is no definitive cure for OLP, and treatment is primarily aimed at symptom control, reducing relapses, and preventing complications. Pharmacological treatment is generally indicated for symptomatic or clinically active disease, particularly atrophic, erosive, or ulcerative forms, whereas management of asymptomatic lesions may rely on periodic surveillance and non-pharmacological measures. In addition, factors such as systemic comorbidities, psychological status, oral hygiene, and specific harmful habits may influence the clinical course of the disease and adherence to follow-up.

In this context, the time intervals between the onset of the first signs or symptoms, the initial consultation, referral to a specialist, and diagnostic confirmation may influence the initiation of follow-up and subsequent clinical management. However, published studies show considerable heterogeneity in the definition and measurement of these intervals, as well as in the attribution of potential delays to the patient, healthcare professionals, or the healthcare system itself [5,6]. In oncology, this lack of uniformity led to the development of the Aarhus Statement, a conceptual framework proposed for standardising the time point milestones of the diagnostic process [7]. In oral cancer, this model has been used to study the intervals related to referral, diagnosis, and start of treatment [8,9]. Nevertheless, its application in OPMD remains limited, despite these lesions also presenting significant diagnostic delays and complex healthcare trajectories.

The aim of the study was to analyse the diagnostic and follow-up intervals of patients with oral lichen planus and to identify clinical and healthcare factors associated with diagnostic timing and continuity of clinical follow-up.

## 2. Methodology

### 2.1. Study Design

A retrospective cohort observational study was conducted, based on the review of the medical histories of patients diagnosed with oral lichen planus, who were treated at the Oral Medicine Unit of the University Dental Clinic of Murcia, in the last 5 years.

The study was approved by the Ethics Committee on 12 November 2025 (ACTA26/2025/CEI), following the principles of the Declaration of Helsinki [10]. The study was designed according to the recommendations from the STROBE guidelines [11].

### 2.2. Participants

A total of 220 medical records of patients with oral lesions compatible with OLP were consecutively reviewed, to which the following inclusion and exclusion criteria were applied:

The patients who met the following criteria were included in the study:

1. Age equal to or higher than 18 years old.
2. Clinical and histopathological diagnosis of oral lichen planus, established according to van der Meij and van der Waal (2003) [12].
3. Availability of complete information on the diagnosis process in the last 5 years, including the date of the first consultation, the biopsy date, the date of diagnostic confirmation, and the date of the last visit recorded in the service.

Those cases that met the following criteria were excluded:

1. Patients with incomplete or missing clinical records regarding diagnosis dates, biopsy report, diagnostic confirmation, or follow-up.
2. Patients without histopathological confirmation available in the archive.
3. Cases diagnosed as oral lichenoid lesion/reaction, lupus erythematosus, leukoplakia, graft-versus-host disease, or other conditions mimicking oral lichen planus.
4. Duplicate records or repeated consultations corresponding to the same patient.
5. Patients whose diagnosis was made outside the study period or whose first consultation occurred before the defined retrospective window.

After the application of the inclusion and exclusion criteria, a total of 21 cases were excluded; as a result, the final study cohort was composed of 199 patients.

A non-probabilistic sampling of consecutive cases was performed, which included all the patients available in the medical archives during the study period who met the inclusion criteria, without the intervention of additional clinical considerations or subjective criteria in the selection of the cases. The study variables were structured into three blocks: demographic characteristics and toxic habits, clinical information, and aspects related to the diagnostic, care, and follow-up process.

#### 2.2.1. Sociodemographic Variables and Toxic Habits

The demographic variables that were collected were age and sex. The toxic habits were collected according to what was reported by the patient and documented in their medical record, including the consumption of alcohol (no consumption, occasional, or habitual), and smoking habit (non-smoker, ex-smoker, or smoker, categorised according to the number of daily cigarettes per day (>10 or <10)).

#### 2.2.2. Clinical Variables

The medical signs and symptoms were recorded based on the information documented during the first visit to our unit. The pain intensity was assessed through a 0 to 10 numerical scale, in which 0 corresponded to the lack of pain, and 10 to the worst pain imaginable.

The clinical form was recorded at the diagnosis (reticular, erosive, mixed, or plaque), according to the predominant clinical presentation documented [13]. In addition, the extent and severity of the disorder were assessed using the Oral Disease Severity Score (ODSS) [14].

The number of professionals consulted before the assessment at our unit was also recorded, along with the treatments received before the final diagnosis. These were classified as: antiseptic, antifungal, topical corticoids, others, and no treatment.

With respect to the follow-up with therapeutic management after the diagnosis, the type of treatment established by the unit responsible was recorded. The patients were classified into three groups: follow-up without active treatment, non-pharmacological treatment (phytotherapeutic), and pharmacological treatment. The follow-up without active treatment consisted of periodic check-ups, which included clinical assessment, the extent of the lesions, the monitoring of oral hygiene, and a photographic record of the changes. The non-pharmacological treatment included conservative measures based on topical use of products, such as phytotherapeutics, aloe vera, or hyaluronic acid. On their part, the pharmacological treatment included the prescription of drugs indicated for the management of the disease, according to the habitual clinical practice depending on the lichen form and extent [15,16]. Given the fluctuating course of OLP, management could vary between visits according to disease activity. For analytical purposes, each patient was therefore assigned to the management category most frequently recorded across follow-up visits.

Patients were managed according to the Unit’s established clinical criteria for OLP diagnosis, assessment, treatment, and follow-up. Clinical and histopathological findings were interpreted using predefined diagnostic and severity frameworks, and clinically ambiguous cases were discussed within the Oral Medicine Unit and resolved by consensus.

#### 2.2.3. Variables of the Diagnostic and Follow-Up Process

The time point analysis was performed following the recommendations from the Aarhus Statement [7] for the definition of the time points and the diagnostic process intervals. All the time points were extracted from the medical history in calendar date format.

The following time points were considered:

- **Date of first symptom**, defined as the moment in which a change in the oral mucosa was identified for the first time, with or without symptoms, either perceived by the patient or detected accidentally by a health professional.
- **Date of the first consultation in primary care**, the first healthcare visit in which, given the characteristics of the lesion, it was possible to start a diagnostic or referral process.
- **Date of first consultation at the oral medicine unit**, date of the first assessment after the referral of the patient to the Unit of Oral Medicine.
- **Date of diagnosis**, defined as the date of diagnostic confirmation based on the clinical-pathological correlation recorded in the medical history.
- **Last recorded visit**, defined as the last recorded consultation date documented in the medical system after the diagnosis.

Based on these time points, the following intervals were defined, initially calculated in calendar days as the difference between two calendar dates:

- **Patient interval**: length of time between the first symptom and the first consultation.
- **Primary care interval**: length of time between the first consultation and the first consultation in the unit of oral medicine.
- **Diagnostic interval**: length of time between the first consultation in the unit of oral medicine and the diagnosis.
- **Total interval**: length of time between the first symptom and the diagnosis.
- **Follow-up interval**: length of time between the date of diagnosis and the last recorded visit.

Given the retrospective character of the study, not all the time points before the assessment at the unit of oral medicine were available for all the patients. For this, each interval was calculated only in the patients who had the two time points necessary for its estimation. To ease the medical interpretation, these were expressed as months in the descriptive analysis and the presentation of the results.

**Figure 1:**
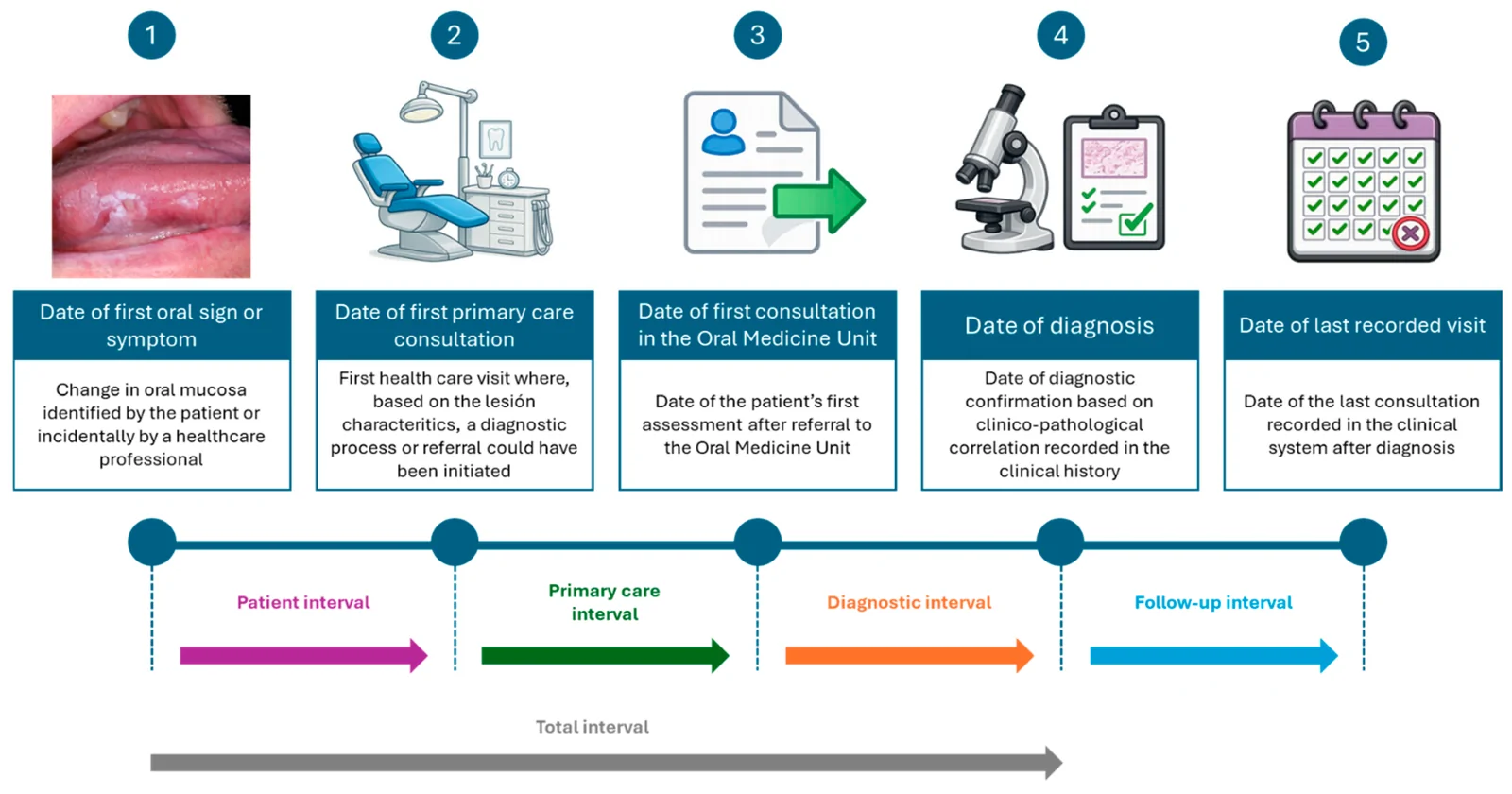
Schematic representation of the time points, the diagnostic process intervals, and the follow-up interval.

The definition of the time points and the derived intervals is shown in Figure 1.

### 2.3. Statistical Analysis

The statistical analysis was performed with the R software (version 4.4.1). The qualitative variables were described through frequencies and percentages, and the quantitative ones through medians and ranges. The diagnostic intervals were analysed through generalised linear models with a quasi-Poisson distribution and logarithmic link function, including the medical and care variables of interest as the predictors. The results were expressed as ratios of means and 95% confidence intervals. The overall significance was assessed through type II contrasts, and post hoc comparisons with Tukey’s corrections were performed. The follow-up interval was analysed through Cox proportional hazards multivariate models, with the proportional hazards assumption being evaluated using Schoenfeld residuals. A value of *p* < 0.05 was considered statistically significant. The analysis was performed through a complete cases approach for each model, so that the effective sample size varied as a function of the availability of the data.

## 3. Results

### 3.1. Descriptive Analysis

The descriptive analysis of the qualitative variables is shown in Table 1. With respect to the quantitative variables, the median age was 60 years (24–90), the intensity of pain was 3 (0–8), and the ODSS index was 4 (1–11).

The effective sample size varied across interval-specific analyses according to the availability of the time points required for each interval. Complete data were available for 177 patients (88.9%) for the patient interval, 151 (75.9%) for the primary care interval, 199 (100%) for the diagnostic interval, and 177 (88.9%) for the total interval. Accordingly, missing interval data affected 22 patients (11.1%), 48 patients (24.1%), 0 patients (0%), and 22 patients (11.1%), respectively.

### 3.2. Patient Interval

After multivariate adjustment, female sex was independently associated with a shorter patient interval compared with male sex (IRR = 0.44; 95% CI: 0.21–0.91; *p* = 0.03). Likewise, age was associated with an increase in the patient interval, with an increase of 4% observed for each additional year of age (IRR = 1.04; 95%CI: 1.01–1.08; *p* = 0.02). In the type II global contrasts, only sex (*p* = 0.03) and age (*p* = 0.01) showed a significant association with the patient interval, while the consumption of alcohol (*p* = 0.11), smoking, (*p* = 0.27) medical form of lichen (*p* = 0.77), number of previous health professionals (*p* = 0.08), previous treatments (*p* = 0.71), pain intensity (*p* = 0.30), and ODSS (*p* = 0.16), did not reach statistical significance (*p* > 0.05).

### 3.3. Primary Care Interval

The analysis of the type II global contrasts of the non-specialised care multivariate model (*n* = 151) showed a significant association with sex (*p* = 0.03), the lichen form (*p* < 0.01), age (*p* = 0.01) and the extent of the clinical activity (*p* < 0.01) (Table 2). After the application of the fitted multivariate model, it was observed that the non-specialised care interval was higher in women (4.6 months) than in men (1.5 months). With respect to the form of lichen, the mixed (1.8 months) and reticular (2.4 months) forms showed significantly shorter intervals than the erosive form (8.3 months), while the plaque form showed a trend in the same direction, but without reaching statistical significance (1.4 months). The post hoc analysis with Tukey adjustment confirmed differences between erosive lichen and the mixed (*p* < 0.01) and reticular (*p* = 0.02) forms, without differences in the remaining comparisons (Table 3). As for the continuous variables, age was associated with a 4% increase in the interval per additional year (IRR = 1.04; 95% CI: 1.01–1.08; *p* = 0.02), while the extent of clinical activity (ODSS) was associated with an increase of 27.7% per additional unit (IRR = 1.28; 95% CI: 1.09–1.50; *p* < 0.01).

### 3.4. Diagnosis Interval

The analysis of the type II global contrasts of the multivariate model of the diagnostic interval (*n* = 199) showed a single significant association between the diagnostic interval and the lichen form (F = 3.77; *p* = 0.01). No significant associations were observed in sex, the consumption of alcohol or tobacco, the number of professionals previously consulted, the previous treatments, age, the intensity of pain, or the extent of the clinical activity (all *p* > 0.05).

After applying the adjusted multivariate model, it was observed that the diagnostic interval was greater in erosive lichen (4.6 months) than mixed (1.6 months), plaque (1.7 months), and reticular (1.7 months) forms (Table 4). The post hoc analyses with Tukey’s adjustment confirmed differences between erosive lichen and the mixed and reticular clinical forms (*p* = 0.01), without differences between the non-erosive forms (Table 5).

### 3.5. Total Interval

The analysis of the global type II contrasts of the multivariate total interval model (*n* = 177) showed statistically significant associations between the total interval and alcohol consumption (*p* < 0.001), the tobacco habit (*p* = 0.03) and age (*p* < 0.001). Sex showed a tendency towards significance (*p* = 0.06). After applying the adjusted multivariate model, it was observed that patients with a daily consumption of alcohol presented longer intervals (55.7 months) than non-consumers (17.1 months) and occasional consumers (24.2 months). With respect to the tobacco habit, a trend towards longer intervals in smokers of more than 10 cigarettes/day was identified (55.7 months) (Table 6). The post hoc analysis with a Tukey adjustment confirmed differences between daily consumption of alcohol and non-consumption (*p* < 0.001), without differences in the remaining comparisons. In the tobacco habit, differences were observed between less than 10 cigarettes/day smokers (12.5 months) and those who smoked more than 10 cigarettes/day (55.7 months), without differences in the remaining comparisons (Table 7). Age was associated with an increase of 3% in the total interval for each additional year of age (IRR = 1.03; IC 95%: 1.01–1.05; *p* = 0.01).

### 3.6. Follow-Up Interval

In the Cox multivariate analysis, age and the type of treatment were significantly associated with the follow-up interval (Table 8). The patients subjected to non-pharmacological treatments presented a lower risk of loss to follow-up than those under follow-up without active treatment, while in the group under pharmacological treatment, a trend in the same direction was observed, although without reaching statistical significance. In addition, although the global contrast of tobacco habit did not reach statistical significance, those who smoked more than 10 cigarettes/day showed an estimate compatible with a higher risk of loss to follow-up.

The proportional risks assumption was assessed using Schoenfeld residuals. The global test showed a significant deviation (*p* = 0.018), which suggests that some effects may not be constant over time. At the covariate level, indications of deviations from previous medication were observed (*p* = 0.031), as well as the extent–activity relationship (*p* = 0.035), while the remaining variables did not show significant deviations (*p* > 0.05). Given that these variables were not significant in the multivariate model, this finding could be considered a limitation in the interpretation of the results.

## 4. Discussion

The main finding of the present study was that the diagnostic intervals in patients with oral lichen planus were not homogeneously distributed throughout the healthcare process, but were associated with demographic, clinical, and behavioural factors. Age was related to an increase in various intervals, while the clinical form of OLP, especially the erosive one, was associated with higher lengths of time in non-specialised care, as in the diagnostic interval in our unit. In addition, daily consumption of alcohol was associated with a longer total interval.

The analysis of diagnostic intervals has been widely developed in oncology settings, in which the Aarhus treatment model has been consolidated as the main conceptual framework to deconstruct and standardise the different phases of the diagnostic process [17,18]. However, its application outside of the context of cancer is still limited, particularly in the area of the OPMD. In this field, the available evidence has been mainly centred on autoimmune blistering diseases, such as pemphigus vulgaris and pemphigoid [5]. More recently, some work has begun to address diagnostic delay in the context of desquamative gingivitis, including cases of erosive oral lichen planus [6], as well as works specifically centred on oral lichen planus, in which erosive and non-erosive forms were compared [19]. However, these studies, aside from being limited in number, were not designed under a structured conceptual framework. This methodological heterogeneity, especially in the definition of intervals and in the measurement of diagnostic times, hinders the comparability between studies and limits the possibility of extracting consistent conclusions.

In our study, age was associated with an increase in the patient interval, non-specialised care interval, and total interval, which suggests that its effect is not exclusively limited to the individual behaviour of seeking care, but can be expanded to different phases of the healthcare process. This finding is in contrast with previous studies, in which age did not show a significant association with diagnostic delay in OLP patients or with other immune-mediated oral diseases [6,19]. These differences could be explained by variations in the study designs, the sample size, the characteristics of the population included, or the definition of the intervals analysed. From a clinical perspective, older patients may tend to normalise chronic oral symptoms or attribute them to other frequent conditions, such as prosthetic trauma, xerostomia, polypharmacy, or concomitant systemic diseases. In addition, the presence of comorbidities could hinder the prioritisation of oral symptoms and delay both the consultation and the referral process [20].

Our results are in agreement with previous studies showing that women had a shorter patient interval, which suggests an earlier consultation after the appearance of signs or symptoms [21]. However, they showed a longer interval of non-specialised care, perhaps related to a higher diagnostic difficulty in the initial phases and the need for more consultations before the definite referral [19,22,23].

The clinical form of the OLP was one of the most relevant variables in the study. The non-erosive forms were associated with shorter intervals than erosive OLP in both non-specialised care and our study. These findings are in agreement with previous works that observed longer referral and diagnosis times in the erosive forms of OLP [19]. Although it could be expected that the erosive forms would lead to a faster referral, as they are symptomatic, their clinical presentation may overlap with other lesions, which increases the complexity of differential diagnosis. On the contrary, the reticular or plaque forms tend to present more recognisable clinical patterns, which can facilitate diagnostic suspicion and shorten the care process. In line with this finding, the ODSS was associated with a longer non-specialised care interval. This result suggests that a greater extent or clinical activity is not necessarily translated into a faster referral. On the contrary, the more extensive or active forms can generate a greater diagnostic uncertainty in the initial phases, especially when erosive erythematous or painful manifestations predominate. This pattern is coherent with that described by Macken et al., who observed longer referral times in erosive forms of OLP, despite their higher symptomatic load [19]. These findings are particularly relevant because erosive OLP has been associated with a higher risk of malignant transformation, making timely diagnosis and close long-term surveillance especially important in these patients [4,19].

Alcohol consumption was associated with the total interval, with longer delays observed in patients who consumed alcohol daily. The specific evidence about the relationship between alcohol and diagnostic delay in OLP is limited. However, specific habits such as alcohol and tobacco consumption can have an influence on the perception of the symptoms, the search for care, and the interaction with the health system [24].

As opposed to the diagnostic intervals, the follow-up interval represents a longitudinal measurement of healthcare continuity when dealing with a chronic and recurring disease with a potential risk of malignant transformation. Due to the fluctuating course of oral lichen planus and the absence of a definitive curative treatment, periodic clinical follow-up aimed at the management of relapses, therapeutic assessment, and the early detection of suspicious changes is recommended [1,15].

Age and treatment type also showed an association with the follow-up. The patients managed through non-pharmacological interventions presented a lower rate of loss to follow-up than those who were solely subjected to clinical monitoring without active treatment. However, this association should be interpreted as exploratory, because treatment allocation in OLP is driven by disease activity and may change over time. The observed therapeutic categories may therefore reflect different stages of disease evolution and be susceptible to confounding by indication, rather than represent an independent or causal effect of treatment on follow-up continuity.

Many studies have described relevant problems of therapeutic adherence and follow-up in patients with oral lichen planus, especially in relation to the prolonged use of topical steroids [25,26]. In this context, clinical follow-up must not be solely understood as a surveillance strategy for the possible malignant transformation, but also as an opportunity to reinforce healthcare education, the management of stress, oral hygiene, and adherence to periodic revisions [16].

The clinical heterogeneity of oral lichen planus and the absence of universally standardised protocols can condition the variability observed in follow-up and the making of clinical decisions. Likewise, the differences in diagnostic experience between professionals, the coexistence of comorbidities, and the difficulties in accessing specialised care could contribute to prolonging follow-up intervals in specific patients. In this sense, the standardisation of monitoring protocols and the improvement of referral pathways to oral medicine units could contribute to optimising the clinical management of these patients.

This study has some limitations that must be considered when interpreting the results. In the first place, its retrospective design was dependent on the quality of the available medical records, which could have conditioned the precision of some of the data, especially those related to the start of the signs or symptoms and, consequently, affected estimates of the patient and total intervals. Systematic information about potentially relevant variables was not available, such as socioeconomic level or level of education, the distance to the healthcare centre, or comorbidities, psychological burden, and health literacy. In addition, the study was conducted in a single specialised Oral Medicine Unit, and referral pathways, diagnostic timing, and follow-up patterns may differ in other healthcare settings. Likewise, some patients under irregular follow-up or losses during monitoring may not be well represented. From a statistical perspective, the effect of some variables on the loss to follow-up may not remain constant during the entire observation, which partially limits the interpretation of specific results. Alternative approaches, such as models with time-varying coefficients, should be considered in future studies to better characterise these time-dependent effects.

Nevertheless, the study includes a broad cohort of patients with a clinical or histopathological diagnosis of OLP, and applies a structured approach to deconstruct the diagnosis process into differentiated intervals, which allows identifying specific phases of the healthcare path that could be improved.

## 5. Conclusions

The application of a structured framework allows differentially characterising the diagnosis intervals in patients with oral lichen planus, and to identify factors associated with different phases of the delay. Age was related to a longer patient interval, primary care interval, and total interval, while erosive forms were associated with a longer primary care interval and diagnosis interval. Likewise, the habitual consumption of alcohol was related to a longer total interval. These findings show that the diagnostic delay in OLP is a multifactorial phenomenon, conditioned by the characteristics of the patient, the clinical presentation, and the healthcare pathway.

The standardisation of diagnostic intervals can improve the comparability between studies and facilitate the identification of modifiable factors in the healthcare process. Future prospective and multi-centre studies must confirm these findings and assess the usefulness of this approach in other OPMDs.

## Figures and Tables

**Table 1:** Descriptive analysis of qualitative variables.

Qualitative Variables	Frequency (%)
Sex	
Male	53 (26.6%)
Female	146 (73.4%)
Alcohol consumption	
Daily	19 (9.5%)
No	151 (76%)
Occasional	29 (15%)
Tobacco consumption	
Ex-smoker	23 (12%)
Smokes < 10	17 (8.5%)
Smokes > 10	20 (10%)
Non-smoker	139 (70%)
Lichen form	
Erosive	15 (7.5%)
Mixed	62 (31.2%)
Plaque	25 (12.6%)
Reticular	97 (48.7%)
Number of previous professionals	
1	36 (18.1%)
2	136 (68.3%)
≥3	27 (13.6%)
Previous treatments	
Antiseptic	29 (14.6%)
Antifungal	23 (11.6%)
Topical corticoids	21 (10.6%)
Others	8 (4%)
None	118 (59.3%)
Post-diagnostic follow-up and therapeutic management	
Follow-up without active treatment	72 (36%)
Non-pharmacological treatment	24 (12.1%)
Pharmacological treatment	103 (1.8%)

**Table 2:** Multivariable quasi-Poisson model for the non-specialised care interval. Bold values indicate statistically significant results (*p* < 0.05).

Variable	IRR (95% CI)	*p*-Value
Sex		
Male	—	—
Female	3.09 (1.12, 10.2)	**0.04**
Lichen form		
Erosive	—	—
Mixed	0.21 (0.09, 0.49)	**<0.01**
Plaque	0.16 (0.02, 0.89)	0.05
Reticular	0.29 (0.13, 0.66)	**<0.01**
Age	1.04 (1.01, 1.08)	**0.02**
ODSS	1.28 (1.09, 1.50)	**<0.01**

**Table 3:** Post hoc pairwise comparisons of lichen form for the non-specialist care interval ** Tukey correction for multiple comparisons. Bold values indicate statistically significant results (*p* < 0.05).

Comparison	Mean Ratio	Adjusted *p*-Value **
Erosive/Mixed	4.71	**<0.01**
Erosive/Plaque	6.15	0.22
Erosive/Reticular	3.48	**0.02**
Mixed/Plaque	1.31	0.99
Mixed/Reticular	0.74	0.88
Plaque/Reticular	0.57	0.92

**Table 4:** Adjusted mean ratios of the multivariable quasi-Poisson model for the diagnostic interval. Bold values indicate statistically significant results (*p* < 0.05).

Variable Lichen Form	IRR (95% CI)	*p*-Value
Erosive	—	—
Mixed	0.34 (0.17, 0.66)	**<0.01**
Plaque	0.36 (0.14, 0.90)	**0.03**
Reticular	0.36 (0.19, 0.69)	**<0.01**

**Table 5:** Pairwise post hoc comparisons of lichen form for diagnostic interval. Bold values indicate statistically significant results (*p* < 0.05).

Comparison	Mean Ratio	*p*-Value
Lichen form		
Erosive/Mixed	2.97	**0.01**
Erosive/Plaque	2.76	0.14
Erosive/Reticular	2.78	**0.01**
Mixed/Plaque	0.93	1.00
Mixed/Reticular	0.93	1.00
Plaque/Reticular	1.01	1.00

**Table 6:** Adjusted mean ratios of the multivariable quasi-Poisson model for the total interval. Bold values indicate statistically significant results (*p* < 0.05).

Variable	Mean Ratio	95% CI	Mean Ratio
Alcohol consumption			
Daily	—	—	—
No	0.31	0.16, 0.61	**<0.01**
Occasional	0.44	0.20, 0.93	**0.03**
Toxic habit smoking			
Ex-smoker	—	—	—
Smokes < 10	0.51	0.18, 1.33	0.20
Smokes > 10	2.29	1.00, 5.41	0.06
No	1.60	0.74, 3.70	0.30
Age	1.03	1.01, 1.05	**0.01**

**Table 7:** Pairwise post hoc comparisons of the total interval. Bold values indicate statistically significant results (*p* < 0.05).

Comparison	Mean Ratio	Mean Ratio
Alcohol consumption		
Daily/No	3.25	**<0.01**
Daily/Occasional	2.30	0.08
No/Occasional	0.71	0.48
Toxic habit smoking		
Ex-smoker/Smokes < 10	1.95	0.55
Ex-smoker/Smokes > 10	0.44	0.21
Ex-smoker/No	0.63	0.66
Smokes < 10/Smokes > 10	0.22	**0.03**
Smokes < 10/No	0.32	0.13
Smokes > 10/No	1.44	0.74

**Table 8:** Multivariate analysis using the Cox model of factors associated with loss to follow-up. Bold values indicate statistically significant results (*p* < 0.05).

Predictor	HR (95% CI)	Global *p*-Value
Sex		0.20
Male	—	
Female	0.78 (0.52, 1.18)	
Alcohol consumption		0.90
Daily	—	
No	1.15 (0.59, 2.24)	
Occasional	1.22 (0.58, 2.58)	
Tobacco consumption		0.08
Ex-smoker	—	
Smokes < 10	1.59 (0.74, 3.41)	
Smokes > 10	2.48 (1.15, 5.39)	
Non-smoker	1.14 (0.62, 2.10)	
Lichen form		0.60
Erosive	—	
Mixed	0.99 (0.49, 2.02)	
Plaque	1.43 (0.59, 3.47)	
Reticular	1.25 (0.61, 2.54)	
Number of previous professionals		0.20
1	—	
2	0.95 (0.60, 1.49)	
≥3	1.51 (0.83, 2.75)	
Previous treatments		0.60
Antiseptic	—	
Antifungal	1.17 (0.63, 2.19)	
Topical corticoids	1.31 (0.71, 2.43)	
None	1.27 (0.78, 2.08)	
Others	0.68 (0.24, 1.93)	
Post-diagnostic follow-up and therapeutic management		**<0.001**
Controls	—	
Non-pharmacological treatment	0.31 (0.16, 0.60)	
Pharmacological treatment	0.71 (0.49, 1.04)	
Age	1.02 (1.00, 1.04)	**0.01**
Pain intensity	1.03 (0.94, 1.12)	0.60
ODSS	1.00 (0.91, 1.11)	>0.9

## Data Availability

The data supporting the findings of this study are available from the corresponding author upon reasonable request. The data are not publicly available due to privacy and ethical restrictions.
